# Morphometric analysis of the palatine triangle in adult human skulls: a potential aid for anthropologists and intraoral bone grafts

**DOI:** 10.1007/s12024-023-00577-7

**Published:** 2023-01-20

**Authors:** Naveen Kumar, Murlimanju BV, Ravi Bhaskar, Ashwini P Aithal, Mohandas KG Rao, Kumar MR Bhat

**Affiliations:** 1 Department of Anatomy, Ras Al Khaimah College of Medical Sciences, RAK Medical & Health Sciences University, Ras Al Khaimah, UAE; 2 Department of Anatomy, Kasturba Medical College, Mangalore, Manipal Academy of Higher Education, Manipal, Karnataka India; 3 Department of Anatomy, Sapthagiri Institute of Medical Sciences & Research Centre, Bengaluru, Karnataka India; 4 Division of Anatomy, Department of Basic Medical Sciences, Manipal Academy of Higher Education, Manipal, Karnataka 576104 India

**Keywords:** Hard palate, Morphometrics, Anthropology, Palatometry, Identification

## Abstract

Morphometrics of the hard palate is an important aspect of forensic anthropology and odontology. Palatine triangle is a triangular area in the hard palate formed by the palatine processes of the maxillae, which can aid intraoral bone grafts. We present the osteological measurements of the palatine triangle (maxillary palate) based on sex, compare it with other hard palate parameters, and establish the correlation between them. Seventy-seven male skulls and 36 female skulls were examined. Various morphometric measurements of the hard palate and palatine triangle were performed meticulously. Mean and standard deviation of each parameter were computed for groups using SPSS 16.0. Relationships between all parameters were analyzed using Pearson’s rank correlation test. The mean palatine length was 38.84 ± 3.75 mm in males and 37.22 ± 4.12 mm in females; the mean palatine breadth was 31.36 ± 2.61 mm in males and 29.78 ± 3.07 mm in females. The mean area of the palatine triangle was 600.88 ± 80.16 mm^2^ in male skulls and 547.96 ± 94.28 mm^2^ in the female skulls. Statistically significant difference in various measurements of the palatine triangle and hard palate between the male and female skulls was noted. Leptostaphyline (narrow palate) was the most prominent type of palate. The area of the palatine triangle showed a strong positive correlation between the total length and breadth of the palate for both male and female skulls. A strong positive correlation was also observed between the palate length and the palatine triangle length. Palatine index and palate breadth had a statistically significant moderate linear relationship. The maxillary palate length, breadth, and area of the palatine triangle were higher in males when compared to females in South Indian origin skulls. Most of the skulls had a narrow palate. The results of this metric analysis of the palatine triangle may lead to a new concept of anatomical research into studying the hard palate, which can be used for sexual dimorphism.

## Introduction

Morphometrics involves the measurement of structures and shapes. It has diverse applications in different scientific fields [1]. Morphometric evaluation of many bony structures of the human body has been carried out. Studying the morphometry of the cranial bones, especially the hard palate, is an essential aspect of forensic anthropology and forensic odontology as it plays a vital role in identifying humans and/or sex in mass disasters and criminal cases, even if less human remains or samples are available. The hard palate is formed by the palatine processes of the maxillae anteriorly and the horizontal plates of the palatine bone posteriorly [2]. These bones meet at a cruciform suture, the intersection of intermaxillary, interpalatine, and palatomaxillary sutures.

**Fig. 1:**
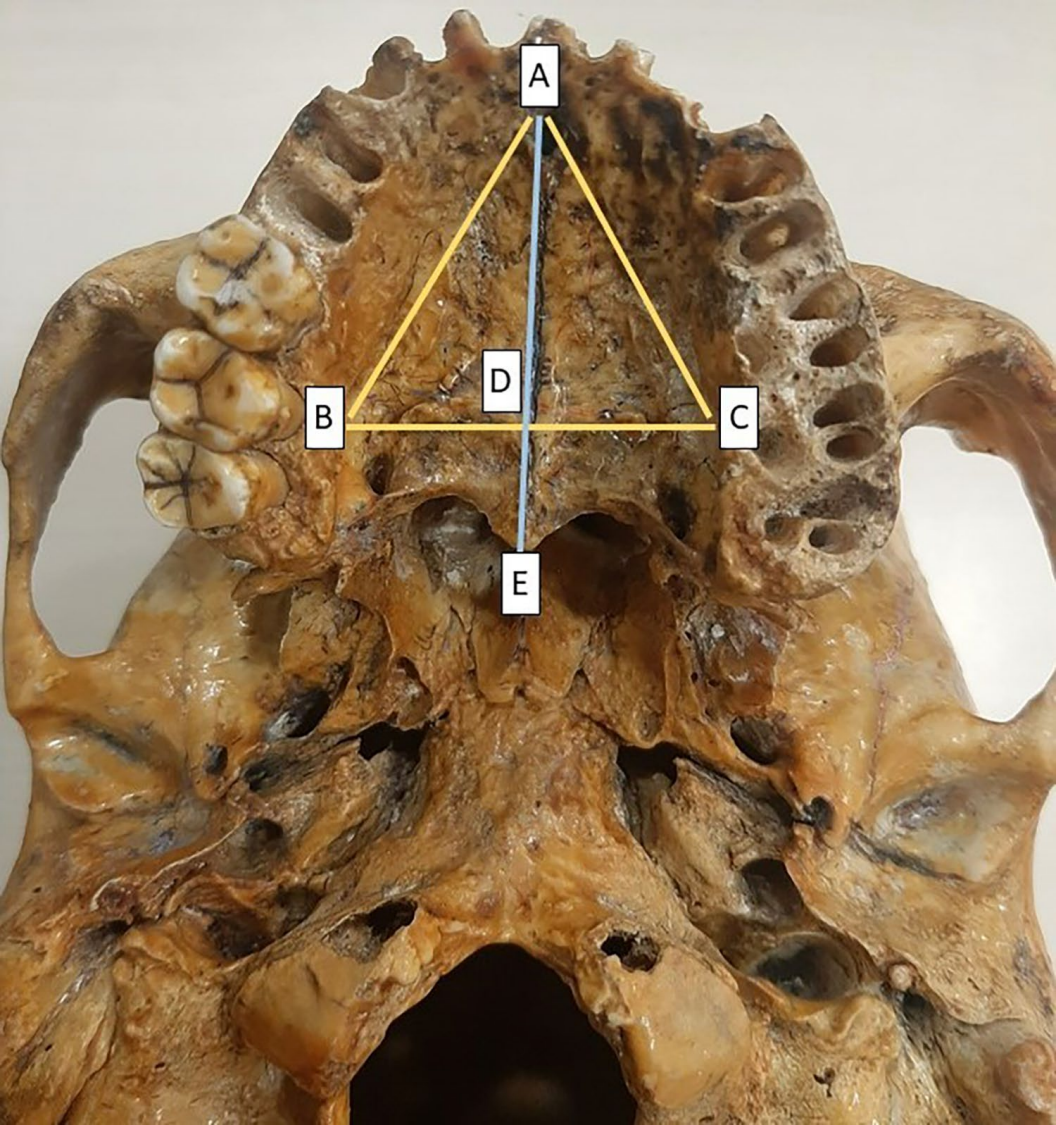
Showing various morphometric measurements taken on the hard palate. **AE**: total length of hard palate; **AD**: length of palatine triangle; **BC**: total breadth of hard palate; **AB**, **AC**: sides of palatine triangle

Although many studies have focused on the various morphometrics of the hard palate [3, 4], there is no data regarding the measurements of the palatine triangle in the human skull. We hypothesize that the shape of the hard palate depends on the triangle’s measurements. Palatine triangle is a triangular area in the hard palate bounded by the greatest transverse diameter of the palate posteriorly and by the lines converging from the extremities of the transverse diameter to the alveolar point (Fig. 1). The palatine processes of the maxillae form this triangle [5]. The palatine triangle is an important component, as an increase in the total length of the hard palate results predominantly from an increase in the anteroposterior dimension of the triangle [6]. A limited number of studies have assessed the palatine process of the maxilla (PPM) as a potential intraoral donor site for reconstructing the oral and maxillofacial region [7]. Morphometrics of the palatine triangle becomes a counterpart of the diameters of the bones which form the hard palate and therefore make it possible to deduce to what extent the palatine processes of the maxilla are engaged in the formation of the hard palate [8].

As the Southern part of India is composed of a heterogeneous population wherein the craniofacial growth is influenced by racial, ethnic, sexual, and dietary differences, standard data of the local population is fundamental in evaluating and diagnosing craniofacial abnormalities [3]. Therefore, this study aims to present the osteological measurements of the palatine triangle based on sex, compare it with other palatal parameters, and establish the correlation between them. Morphological classification of the hard palate was determined by calculating the palatine index, and sexual dimorphism in measurements was also noted, thereby providing reference data of palatal measurements of the South Indian population.

## Materials and methods

The study included a total of 113 dry adult skulls obtained from the Department of Anatomy. This study complies with the Helsinki Declaration and local ethical guidelines. The authors certify that they have obtained the Institutional Ethics Committee clearance for this study. Skulls that were modified and fetal skulls were excluded from the study. All skulls were first examined for sex determination using the standard method [9]. If more traits pointed towards a female, it was taken as a female skull, and the same criteria were applied to male skulls. Seventy-seven male skulls and 36 female skulls were examined. The hard palate was measured using a calibrated rigid ruler and silk suture thread. All measurements of the palate were taken twice, in the same manner, and by the same investigator to avoid interobserver variability. The following measurements were taken and aggregated based on sex (Fig. 1):

1. Maxillary palate breadth (BC): Maximum width of the hard palate at the gingival margin of the maxillary second molar of one side to the other side.
2. Maxillary palate length (AE): Maximum length of the hard palate from the anterior margin of the incisive fossa to the posterior nasal spine.
3. Sides of the palatine triangle (AB, AC): Maximum transverse diameter of the palate from the outer border of second molar teeth (BC) and measurement of the lines converging from ends of transverse diameter to the incisive fossa.
4. Length of the palatine triangle (AD): Maximum length of the hard palate from the anterior margin of the incisive fossa to the midpoint of the maximum width of the palate.
5. Area of the palatine triangle.
6. Palatine index = maximum palatine width (BC)/maximum palatine length (AE) × 100.

Based on the palatine index (%), palates were then categorized as leptostaphyline/narrow palate (≤ 79.9%), mesostaphyline/ medium palate (80–84.95%), and brachystaphyline/broad palate (≥ 85%).

The mean and standard deviation of each parameter were calculated using SPSS v 16.0 (SPSS Inc., Chicago, IL). *P*-value (level of significance) was set at 0.05. Independent *t*-test was performed to compare the various parameters and to determine whether there were any differences between male and female skulls. Pearson’s correlation analysis was used to assess the associations between the measured variables. Results were represented in relevant tables and graphs.

## Results

### Sex differences

There was a statistically significant difference in the measurements of the hard palate and the palatine triangle between the male and female skulls (*p* < 0.05) (Table 1).

**Table 1:** Table showing the various morphometric measurements of the hard palate and the result for independent *t*-test to compare the level of significance between the studied variables in the male and female skulls. *p < 0.05

Variables		*N*	Mean (mm)	Std. deviation	*p* value (between male and female skull)
Palatine length	Male	76	38.84	3.75	0.041*
	Female	36	37.22	4.12	
Palatine breadth	Male	76	31.36	2.61	0.006*
	Female	36	29.78	3.07	
Length of palatine triangle	Male	76	38.31	3.81	0.042*
	Female	36	36.66	4.20	
AB (sides of palatine Triangle)	Male	76	38.63	3.40	0.006*
	Female	36	36.60	3.02	
AC (sides of palatine Triangle)	Male	76	38.19	3.67	0.003*
	Female	36	36.66	2.96	
Area of palatine triangle	Male	76	600.88 mm^2^	80.16	0.003*
	Female	36	547.96 mm^2^	94.28	

### Descriptive statistics

The mean maxillary palatine length was 38.84 ± 3.75 mm in males and 37.22 ± 4.12 mm in females. The mean maxillary palatine breadth was 31.36 ± 2.61 mm in males and 29.78 ± 3.07 mm in females (Table 1).

### Dimensions of the palatine triangle

**Fig. 2:**
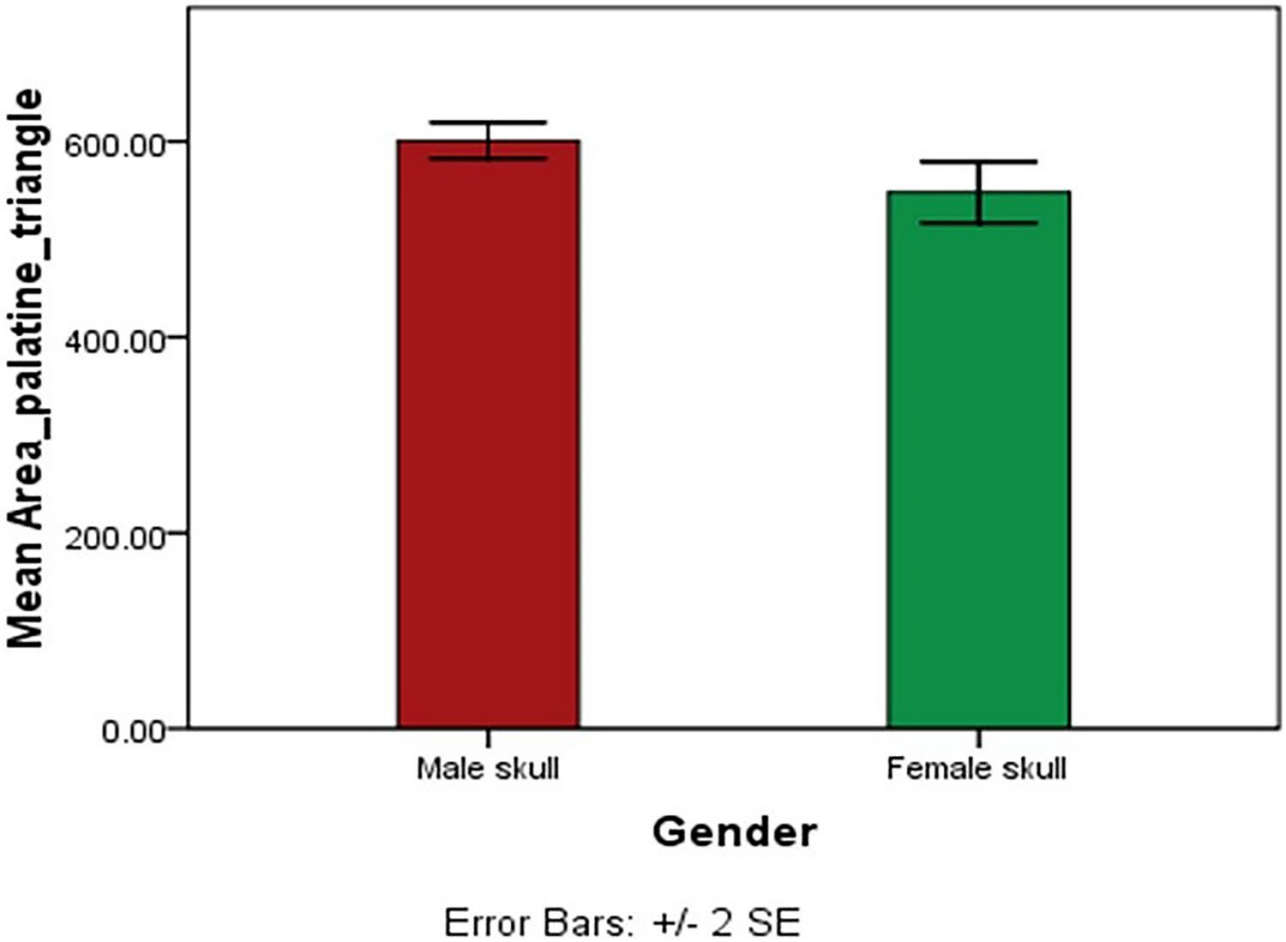
Showing the mean area of palatine triangle (mm.^2^) in male and female skull (*p < 0.05)

Statistical analysis showed significant differences indicating sex differences in various measurements of the palatine triangle between the male and female skulls (Table 1). The mean area of the palatine triangle was 600.88 ± 80.16 mm^2^ in male skulls and 547.96 ± 94.28 mm^2^ in the female skull; the difference was statistically significant (*p* = 0.003) (Fig. 2).

### Types of palate

In the Palatine index (%), the ratio of the length to the breadth was calculated. The values indicated that the leptostaphyline/narrow palate percentage was 47% in male skulls and 55% in female skulls. The percentage of mesostaphyline/medium palate was 22% in male skulls and 14% in female skulls. The percentage of the brachystaphyline/broad palate was 31% in male skulls and 31% in female skulls. It was noted that no statistically significant difference was found in the palatine index among the male and female skulls (*p* > 0.05) (Table 2).

**Table 2:** Showing the frequency of types of palate according to the palatine index in male and female skulls

**Types**	**Palatine index (%)**	**Male skull *N* (%)**	**Female skull *N* (%)**	***p* value**
Leptostaphyline	≤ 79.9	36 (47%)	20 (55%)	*p* > 0.05
Mesostaphyline	80–84.9	17 (22%)	5 (14%)	
Brachystaphyline	≥ 85	24 (31%)	11 (31%)	

### Analysis of correlation

**Fig. 3:**
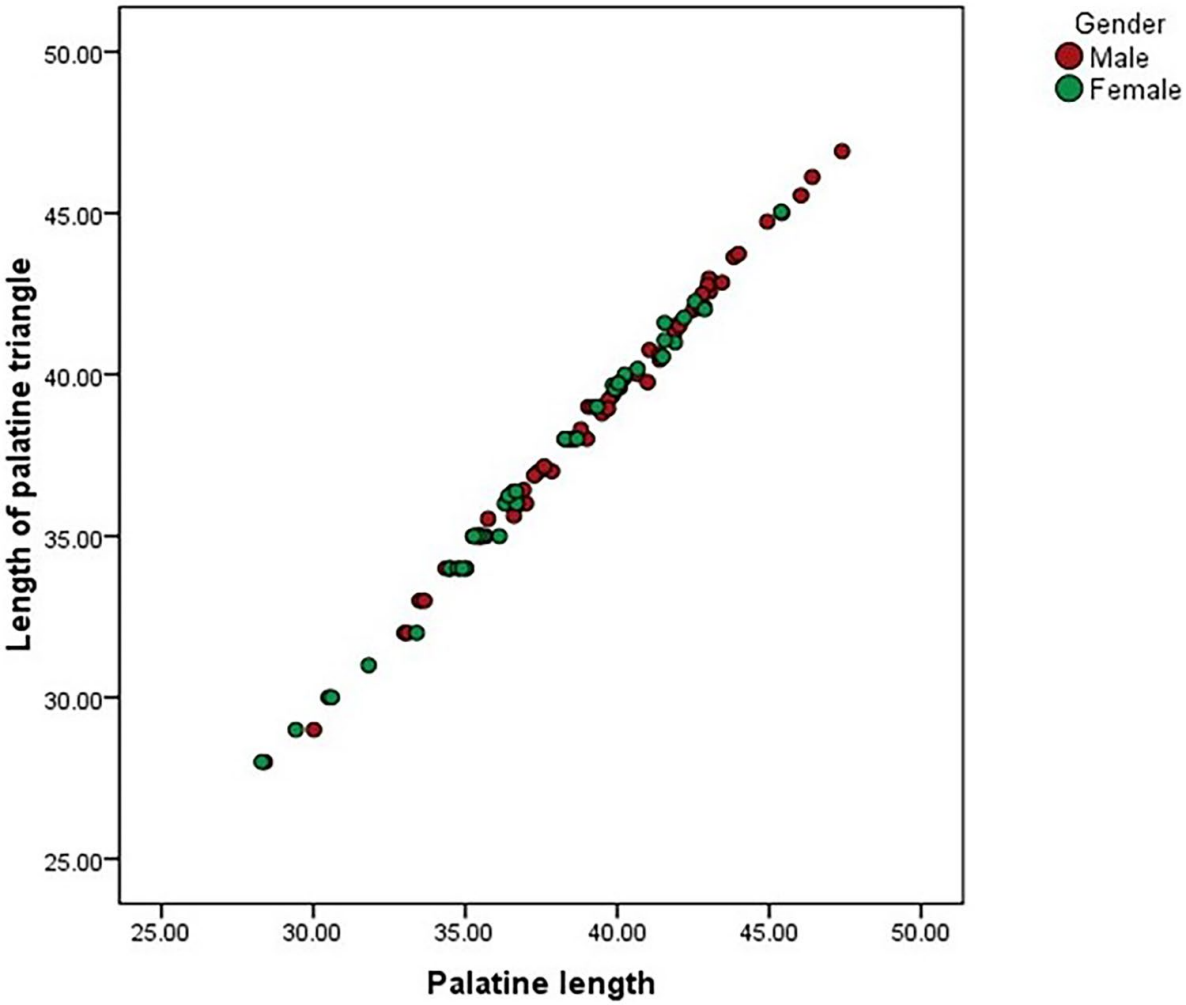
Scatter plot showing the correlation between length of palatine triangle vs. palatine length in male and female skulls

**Fig. 4:**
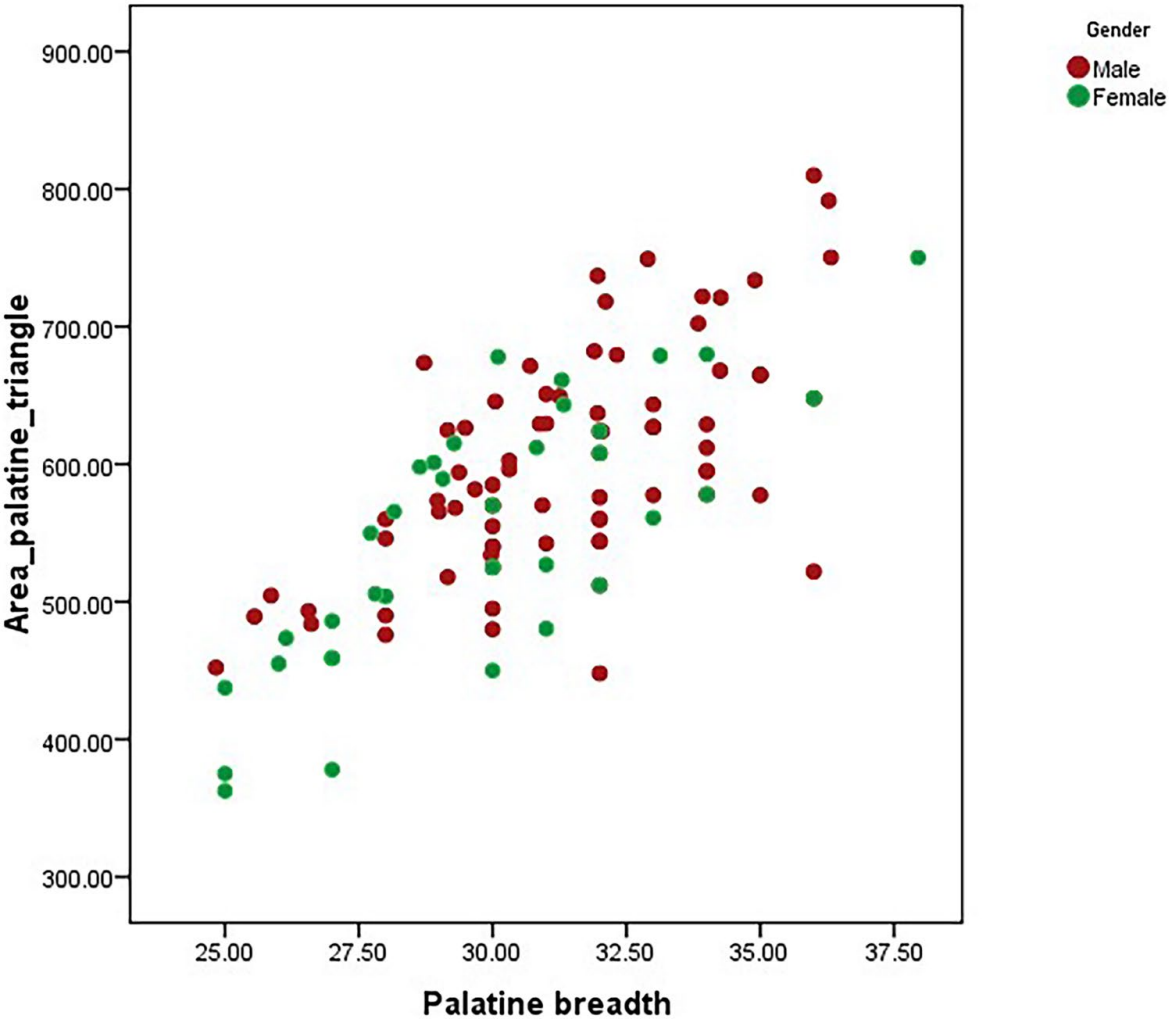
Scatter plot showing the correlation between area of palatine triangle vs. palatine breadth in male and female skulls

**Fig. 5:**
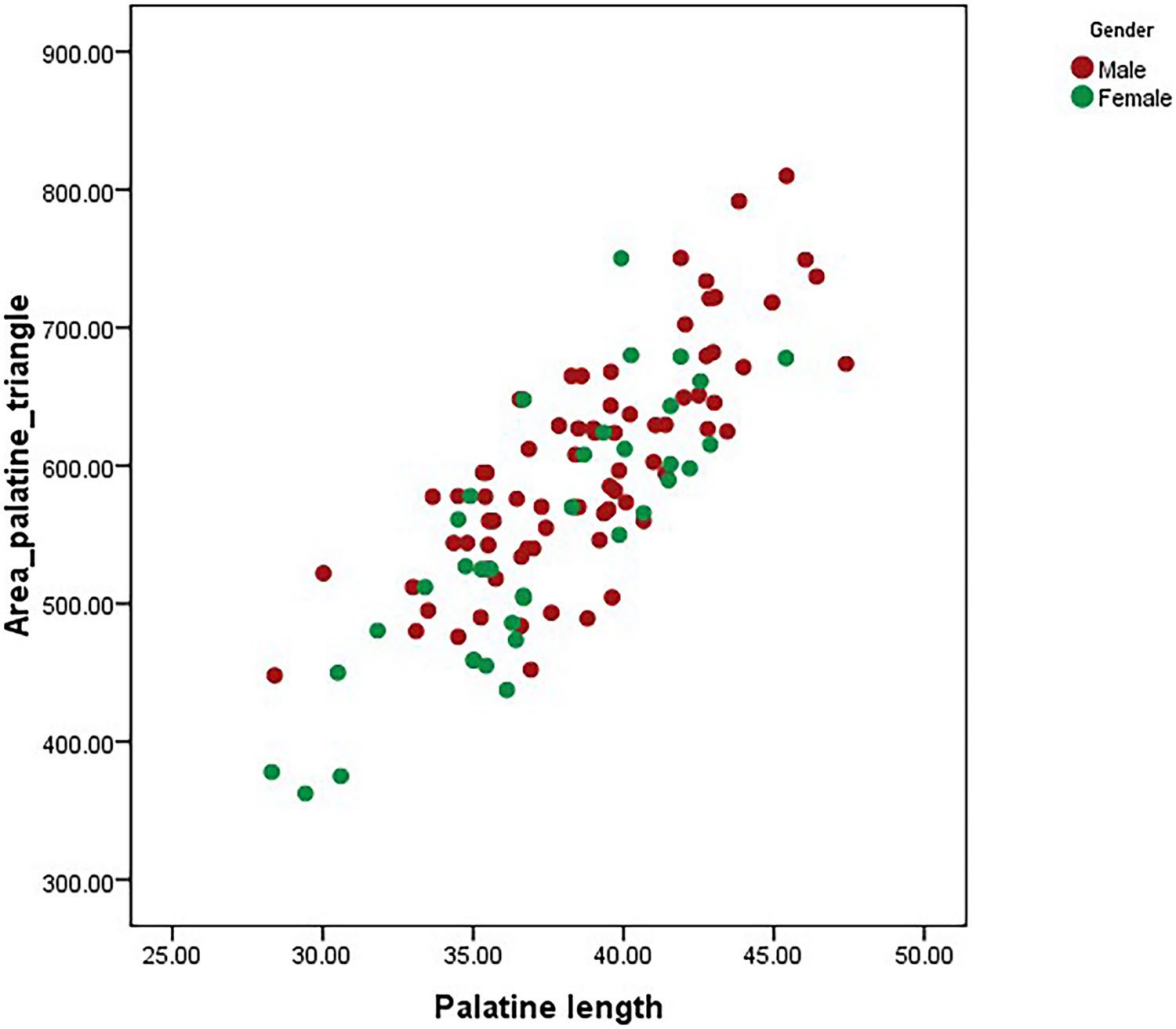
Scatter plot showing the correlation between area of palatine triangle vs. palatine length in male and female skulls

Pearson correlation analysis was performed between the palatal measurements and the palatine triangle. The area of the palatine triangle showed a strong positive correlation between the total length of the maxillary palate (*r* = 0.80; *p* = 0.001) and the total breadth of the maxillary palate (*r* = 0.70; *p* = 0.001) for both male and female skulls (Table 3). A strong positive correlation was also observed between the length of the palate and the length of the palatine process of the maxilla (length of palatine triangle) (*r* = 0.99; *p* = 0.001) (Fig. 3). Based on these results, we can state that area of the palatine triangle and the palate dimensions has a statistically significant linear relationship which is expected, as breadth/length are components of the maxillary palate. This indicates that these variables tend to increase together (Figs. 4 and 5).

**Table 3:** Table showing the correlation between the area of palatine triangle and the various measurements of palate in skulls

Variables	Pearson correlation coefficients (*r*)	*p* value
Area of palatine triangle vs Palatine length	0.80	*p* = 0.001
Area of palatine triangle vs Palatine breadth	0.70	*p* = 0.001
Length of palatine triangle vs Palatine length	0.99	*p* = 0.001

The palatine index and breadth of the maxillary palate had a statistically significant moderate linear relationship (*r* = 0.57; *p* = 0.001). The direction of the relationship is positive, meaning that the shape of the palate is dependent on the breadth of the palate. There was a negative statistically non-significant relationship between the palatine index and the area of the palatine triangle (*r* =  −0.15) (Table 4).

**Table 4:** Table showing the correlation between the Palatine index and the measurements of palate in male and female skulls

Variables	Pearson correlation coefficients *(r)*	*p* value
Palatine index vs Palatine breadth	0.57	*p* = 0.001
Palatine index vs area of palatine triangle	−0.15	*p* = 0.09

## Discussion

The hard palate is preserved even during severe damage to the skull. It is appropriate for studying sexual dimorphism as previous studies have affirmed that palatine length and width are higher in males than in females [10]. Compared to extraoral sites, intraoral donor sites like the palatine process of the maxilla are more advantageous owing to their location, accessibility, size, and type of graft [7, 11]. Most of the studies conducted to date are preliminary clinical and radiological studies on the available bone volume in the palatine bones of the maxilla as an alternate source for intraoral grafts [12]. In only one study on dry skulls, the authors conclude that the anterior palatal region could be a reliable donor site for maxillofacial and oral surgery procedures [13]. The present study, therefore, aims to provide basic osteological measurements of the maxillary palate in the palatine triangle and their correlation with the dimensions of the hard palate.

In our study, we observed that the palatine process dimensions that reflect the palatal size were significantly higher in males than in females, indicating that these parameters are sexually dimorphic. Our findings aligned with previous reports on other populations using various study designs [14–17]. Significant differences in the palatal measurements have been observed in the literature, which can be due to the differential growth patterns of the facial region, genetic background, and ethnicity, and also due to pathologies such as allergies and prolonged mouth breathing [18]. It is very interesting to note that there existed differences in some of the measured parameters of the palate among the same population, which suggests that anatomical variations of the palate might also be present within a population [19]. The observed differences can also be due to the fact that the palate, during its development and life, is subjected to various forces that change its shape, like the forces of tongue muscles/muscles of mastication or the pull of facial muscles. This factor might also affect the concept of using the hard palate’s shape in dry skulls as a diagnostic indicator for sexual dimorphism [20].

Morphometric knowledge of the hard palate is beneficial in various dentistry disciplines like orthodontic procedures and orthognathic treatments. Correlation analysis between the various palatine measurements in the present study shows a positive relationship between these parameters. The area of the palatine triangle showed a strong positive correlation between the length and breadth of the palate for both male and female skulls. A strong positive correlation was also observed between the length of the palate and the length of the palatine process of the maxilla (length of the palatine triangle). Therefore, we can postulate that, as the length and breadth of the palatine processes of maxilla increases, the length and breadth of hard palate also increases. However, no correlation was observed between the shape of the palate and the palatine triangle, suggesting that the measurements of the palatine process do not alter the overall shape of the palate. Morphometrics of the palatine processes seems important to evaluate to what extent the diameters of the bony palate increase with an increase in the length of the palatal process. The palatine triangle could be regarded as a keystone anatomical structure that is engaged in the growth of the hard palate and in turn the development of the whole skull.

In the present study, the computation of the palatal index showed that most of the skulls had narrow (leptostaphyline) palate (47% in male skulls, 55% in female skulls). The percentage of the middle palate (mesostaphyline) was 22% in male skulls and 14% in female skulls. The percentage of the broad palate (brachystaphyline) was 31% in male skulls and 31% in female skulls, with no significant statistical difference between male and female skulls. Other studies reported similar findings [21, 22]. They also opine that around 63% of Indian skulls studied by them had a narrow palate followed by a broad palate. The knowledge of the palatine index is necessary because the palate’s shape has been associated with many syndromes such as Apert syndrome, Turner’s syndrome, Marfan syndrome, and Franceschetti–Treacher–Collins syndrome [23]. Knowledge of palatal indices would be helpful to anatomists, forensic experts, and anthropologists, as the values obtained are of importance to determine the race and gender of the human skull. It is also helpful for orthodontic procedures and surgical repair of the cleft palate, as the anatomy of the maxillary palate may influence the orthodontic expansion and speech after the surgery [24].

Conventionally, the maxilla’s anterior region comprising the maxilla’s palatine processes has been considered a recipient site for bone grafts. In contrast, very few studies have considered it a donor site for intraoral bone grafts [25]. Based on the results of a few pre-clinical and radiology studies, it is apparent that this region of the palate is advantageous and can be reliably selected as a donor site in the oral and maxillofacial reconstructive, implantology, and periodontal regeneration procedures [13]. It has been found that the quantity and quality of bone grafts that can be obtained from the palatine triangle region are similar or even superior to those of bone grafts from other intraoral donor sites. The region of the palatine triangle has also been regarded as a possible donor site for the regeneration of atrophic alveolar defects [26]. Resection of the hard palate can also be done to manage benign and malignant tumors [27].

Limitations of the study include the fact that the present investigation used dry adult human skulls for measurements of the hard palate. Although the primary objective of the study was achieved, further research is needed to answer the many clinical questions, like obtaining the most suitable graft according to the type of defect and minimizing risks and complications during these procedures.

In conclusion, the maxillary palatine length, breadth, and area of the palatine triangle were higher in males when compared to females in South Indian origin skulls. It found that most of the skulls had a narrow palate. This metric analysis of the palatine triangle may lead to a new concept of anatomical research into the study of the hard palate, which can be used for sexual dimorphism and to study craniofacial growth in this population.

## Key points

1. Morphometrics of the hard palate and maxillary palate was performed in South Indian origin adult human skulls.
2. Measurements of the palatine triangle (maxillary palate) were correlated to other measurements of the hard palate.
3. Measurements of the hard palate are useful in sex determination and are one of the key measurements in anthropology.
4. The shape of the hard palate is dependent on the breadth of the hard palate. The length and breadth of the maxillary palate are higher in male skulls than in female skulls.

## Data Availability

All data is available with the authors.
